# *In vivo* micro-scale tomography of ciliary behavior in the mammalian oviduct

**DOI:** 10.1038/srep13216

**Published:** 2015-08-17

**Authors:** Shang Wang, Jason C. Burton, Richard R. Behringer, Irina V. Larina

**Affiliations:** 1 Department of Molecular Physiology and Biophysics, Baylor College of Medicine, Houston, TX 77030, USA; 2 Department of Genetics, University of Texas M.D. Anderson Cancer Center, Houston, TX 77030, USA

## Abstract

Motile cilia in the mammalian oviduct play a key role in reproduction, such as transporting fertilized oocytes to the uterus for implantation. Due to their small size (~5–10 μm in length and ~300 nm in diameter), live visualization of cilia and their activity in the lumen of the oviduct through tissue layers represents a major challenge not yet overcome. Here, we report a functional low-coherence optical imaging technique that allows *in vivo* depth-resolved mapping of the cilia location and cilia beat frequency (CBF) in the intact mouse oviduct with micro-scale spatial resolution. We validate our approach with widely-used microscopic imaging methods, present the first *in vivo* mapping of the oviduct CBF in its native context, and demonstrate the ability of this approach to differentiate CBF in different locations of the oviduct at different post-conception stages. This technique opens a range of opportunities for live studies in reproductive medicine as well as other areas focused on cilia activity and related ciliopathies.

Cilia are microtubule-based organelles located on the surface of a variety of epithelial cells. Motile cilia are present in the organs in the body including oviduct, lungs, brain and airway, and act to propel fluid or mucus^1^,^2^. The mechanisms of cilia motility are complex and have not been fully understood^3^,^4^,^5^, though its associated abnormalities, such as primary ciliary dyskinesia, often result in a wide range of diseases^6^,^7^. The mammalian oviduct is one of the organs that rely heavily on the motility of cilia for its normal functions. The inner lumen of the oviduct is covered with ciliated epithelial cells^8^. With the size of ~5–10 μm in length and ~300 nm in diameter, the oviduct cilia beat periodically to support the mammalian reproduction, such as regulating spermatozoa in the fertilization process^8^,^9^,^10^. The transport of fertilized oocytes to the uterus for implantation is facilitated by the cilia as well as by muscular contractions in the oviduct wall^8^,^9^,^10^. The cilia beat frequency (CBF) is widely used as a fundamental measure of the cilia activity for studying its role in the reproductive process^11^,^12^,^13^,^14^,^15^,^16^.

Bright-field microscopy and confocal microscopy are two major imaging techniques that have been extensively applied for *in vitro* visualization of cilia and assessment of CBF^11^,^12^,^13^,^14^,^15^,^16^,^17^,^18^,^19^,^20^. Relying on the brightness change caused by the beat of cilia, bright-field microscopy generally does not require exogenous contrast agents. The imaging depth required to cover the cross-section of the intact mouse oviduct tube is over 800 μm (larger for other mammalian models and humans). Because of the limited imaging depths of these two methods, during experiments, the oviduct has to be dissected from the body and opened up to expose the ciliated lumen for direct imaging. This might significantly alter the biochemical and biomechanical environment of the cilia and thus lead to inaccurate interpretation of the physiological process^4^,^21^. Detecting periodical variations in the backscattered laser light to measure the CBF have also been previously reported^22^,^23^,^24^. Although *in vivo* application in human fallopian tube has been achieved through an endoscopic fiber optic probe^25^, the approach is limited to a single point integrated measurement, which is lacking both lateral and in-depth information.

Optical coherence tomography (OCT) is a noninvasive depth-resolved 3D imaging modality that provides micro-scale spatial resolution with the imaging depth of 1–2 mm in highly-scattering tissues^26^,^27^,^28^. Recent developments of OCT-based methods for the imaging of cilia dynamics have been focused on the respiratory tract^29^,^30^,^31^,^32^. With micro-OCT (μOCT) that provides 1-micron spatial resolution^33^, Liu, *et al*. successfully imaged and visualized the phases of the ciliary stroke pattern from human bronchial epithelial culture and *ex vivo* swine trachea, thus directly obtaining the frequency of cilia beat^30^. While the size of cilia is below the resolution limit of traditional OCT imaging and the cilia cannot be directly visualized, Oldenburg, *et al*. employed the variations of the speckles produced by the movement of cilia to define the cilia location and measure the CBF^29^. The imaging of the cilia location was conducted on the *in vitro* model of airway epithelium with the mapping of the standard deviation of the speckle change from different frequency bands, and the CBF measurement was demonstrated on the *ex vivo* and *in vitro* mouse trachea samples^29^. Focusing on the cilia-driven fluid flow, Jonas, *et al*. and Huang, *et al*. developed OCT-based particle tracking velocimetry to image the quantitative flow dynamics induced by the cilia on *Xenopus* embryonic model^34^,^35^, which indirectly probes the ciliary function. These studies established the feasibility of using OCT for functional cilia imaging. However, quantitative micro-scale mapping of both cilia location and CBF over a large field of view has not been previously demonstrated. Besides, in all these studies the ciliated surface was directly exposed for imaging.

In this work, we combined live mouse manipulation approach with the further development of the OCT-based speckle-variance cilia-detection method^29^ to introduce a tomographic imaging technique capable of mapping both the cilia location and the CBF in the intact mouse oviduct *in vivo* with micro-scale spatial resolution and depth-resolved field of view that covers the whole depth of the oviduct. Since the method takes advantage of the speckles produced by the interference of light^36^, no exogenous contrast agents are required. As a novel form of functional OCT (fOCT)^37^,^38^,^39^, we utilize both the amplitude and the frequency position of the major peak from the ciliary motion spectrum, and reconstruct, for the first time, high-resolution mapping of both cilia location and CBF through tissue layers *in vivo*. We validate the approach using histological immunofluorescence microscopy for the cilia-location mapping and using bright-field microscopy for the CBF mapping. With this method, we present the first *in vivo* application of the oviduct CBF mapping in relation to the post-conception stages and the anatomical locations. Our results indicate that described fOCT approach can be successfully applied to study the dynamic ciliary behavior in mouse oviduct *in vivo*, opening the door for a variety of live studies of mammalian reproduction and infertility, as well as other research areas involving the analysis of cilia activity.

## Results

### Description of *in vivo* fOCT approach

**Figure 1:**
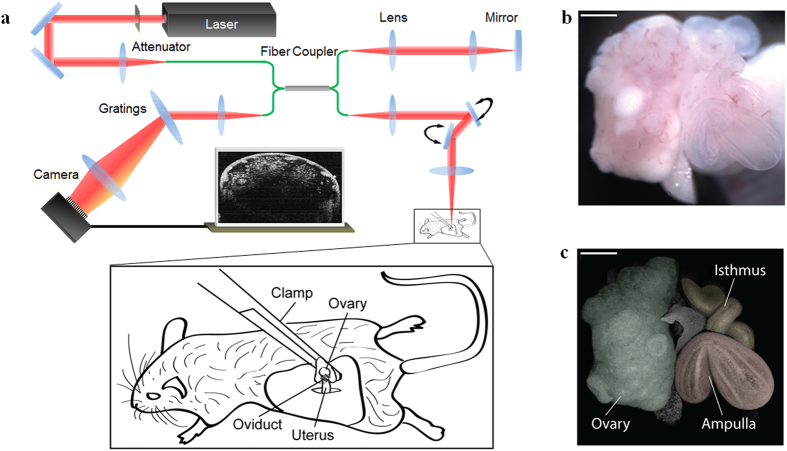
*In vivo* fOCT imaging setup. (**a**) The experimental setup for *in vivo* mouse oviduct imaging. (**b**) Microscopic image of the mouse reproductive organs exposed for fOCT imaging. (**c**) 3D OCT structural image of the same area showing overall morphology of the mouse ovary and oviduct. Distinct regions of the oviduct (ampulla and isthmus) are color-coded. Scale bars correspond to 600 μm. The schematic in (**a**) was created by the authors using Adobe Illustrator software.

Our fOCT imaging technique is based on a spectral domain OCT system (Fig. 1a) which consists of a near-infrared broad-band laser source, a fiber-based Michaelson interferometer and a high-resolution spectrometer^40^. For fOCT data acquisition, the OCT system was set to acquire a time sequence of B-scans at each 3D frame location to capture the dynamics of speckles. The frame rate was set to be 100 Hz with 1024 frames acquired for each position. According to the Nyquist-Shannon sampling theorem, the maximum measurable frequency with these settings is 50 Hz, which, based on the previously-reported values of below 25 Hz, is sufficient to resolve the CBF in mammalian oviduct^12^,^13^,^14^,^15^,^41^,^42^. Also, the resulted spectral resolution is ~0.1 Hz for the frequency measurement and the time required for the fOCT imaging of each frame location with these settings is 10.24 seconds.

For the imaging of the mouse oviduct *in vivo*, a small incision (~1 cm long) was made parallel to the dorsal midline on one side of the anesthetized female. The reproductive organs (the ovary, oviduct, and a part of the uterine horn) were gently pulled out through the incision with a forceps and stabilized with a vessel clamp for imaging (Fig. 1a). The experimental stabilization approach (detailed in the Methods section) helps to prevent the tissue motions associated with animal breathing and heartbeat. Similar procedures for exposing reproductive organs are routinely used to transfer zygotes in the production of transgenic mice^43^; these procedures do not compromise animal survival and are not associated with reproductive complications. Figure 1b shows a microscope image of the exposed reproductive organs. For comparison, Fig. 1c contains a 3D OCT structural image in which distinct morphological features of the oviduct are clearly evident and can be used to locate the positions for the mapping of cilia location and CBF.

### Method for mapping cilia location and CBF with fOCT

**Figure 2:**
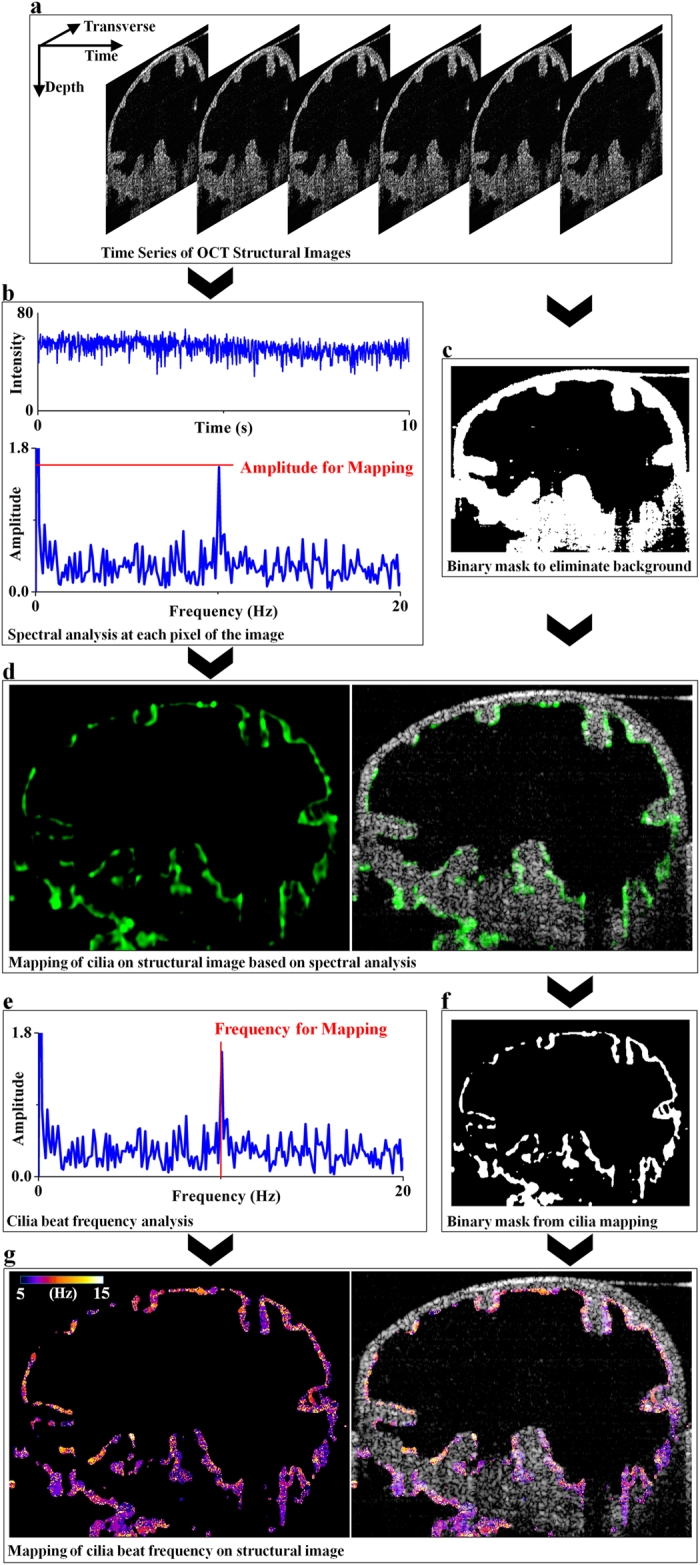
Method for CBF mapping with fOCT. (**a**) Time series of 2D depth-resoled OCT structural images through the mouse oviduct is acquired. (**b**) Typical temporal intensity (speckle) profile from the location of cilia and the corresponding amplitude spectrum from fast Fourier transform. (**c**) Binary mask created from the averaged image of the time series of OCT B-scans. (**d**) Mapping of the peak amplitude from the spectrum indicating the location of the cilia with green color. (**e**) The position of the peak from the obtained spectrum represents the frequency of the cilia beat. (**f**) Binary mask created from the cilia location image. (**g**) Mapping of the frequency of cilia beat to the OCT structural image providing the fOCT image of the ciliary activity. The observed ciliated surface at the lower left portion in (**d**) and (**g**) corresponds to the ciliated grooves in the luminal surface of the adjacent deeper-positioned loop of the oviduct.

The CBF mapping was obtained through spectral analysis of the speckle variation in the continuously recorded B-scan OCT structural images (Fig. 2a). From the recorded image series, 3D matrices with the transverse, depthwise and temporal dimensions were obtained, and the OCT intensities (speckles) over time were analyzed for each spatial pixel. Cilia in the mouse oviduct generally have a length of around 5 μm^44^, and thus the beat of the cilia results in the movement of the scatter (the top part of the cilia) in the scale below the spatial resolution of the OCT system. This type of optical path-length change places the interference of light at different states (e.g. constructive and destructive interference)^45^, which produces variations of the OCT intensity over time at the spatial locations corresponding to cilia (Fig. 2b). Since the beat of cilia in the oviduct is periodic^13^, fast Fourier transform was performed to the temporal intensity profile to obtain the amplitude spectrum of the cilia beat (Fig. 2b). In parallel, a binary mask (Fig. 2c) was produced from the averaged B-scan OCT images over time to eliminate the background and highlight the oviduct tissue. The amplitude spectra from all spatial pixels associated with the oviduct tissue were then plotted together to determine the thresholds for the amplitude and frequency in the spectral domain. Because we do not expect CBF to exceed 25 Hz^12^,^13^,^14^,^15^,^41^,^42^, all signals at the frequency window of 25–30 Hz were taken as noise and were used to set the amplitude threshold for the whole frequency range. All the frequency peaks (except for the zero-frequency components) above this threshold were considered as the CBF signal. The frequency thresholds were set according to the lowest and the highest peak positions (except for the zero-frequency components) in the amplitude spectra. Thus, the peak amplitudes from the frequency spectra were mapped to the structural image of the oviduct, revealing the location of the cilia distributed on the inner lumen (Fig. 2d). The lack of similar periodic motion in the other parts of the oviduct tissue, such as the mucosa, creates the contrast for imaging the cilia location. For the mapping of the CBF, the frequency position of the peak in the amplitude spectrum (Fig. 2e) was obtained for each spatial position of the cilia according to the binary mask (Fig. 2f) produced from the cilia location image. Consequently, the image of CBF mapped to the 2D depth-resolved oviduct structure was generated (Fig. 2g) as the visual representation of the cilia dynamics. For functional imaging in 3D, the generated 2D fOCT images from all the frame locations can be put together to form the 3D map of cilia location and CBF, where tomographic sections from all directions can be easily visualized and analyzed.

### Validation of fOCT mapping of cilia location

**Figure 3:**
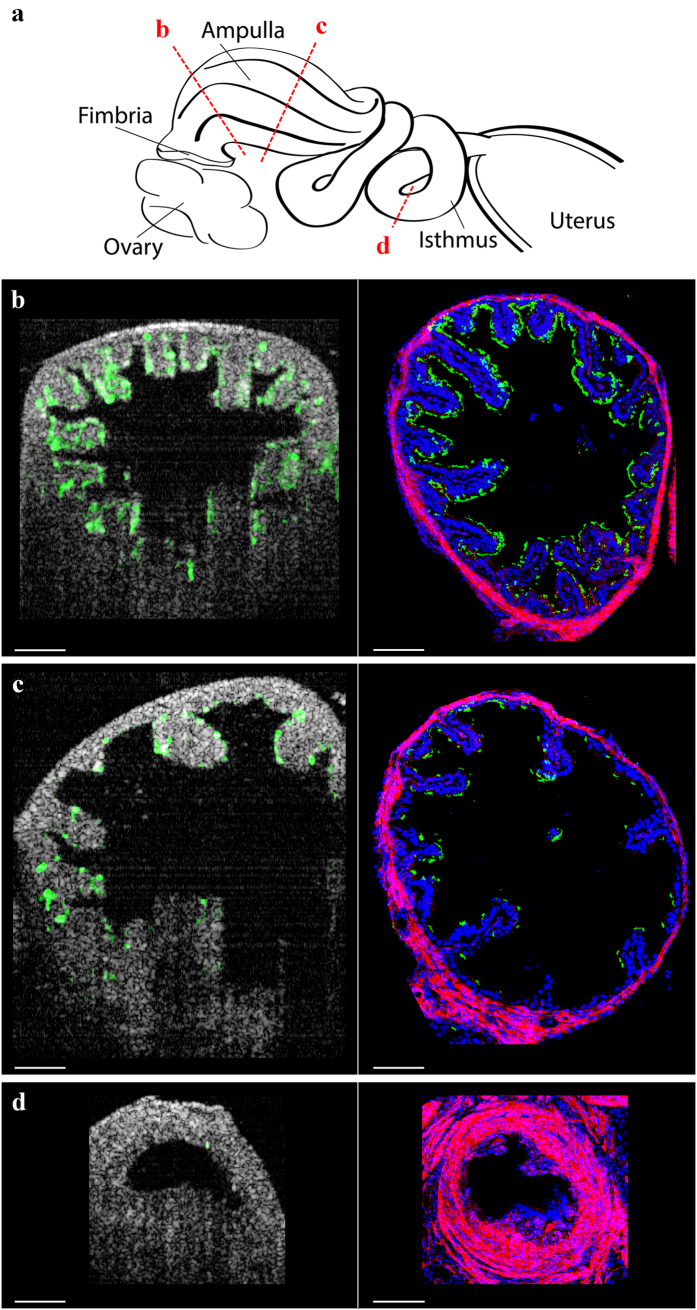
Validation of fOCT mapping of cilia location. (**a**) Illustration of the positions imaged and analyzed from the ampulla and isthmus of the mouse oviduct. (**b**–**d**) *In vivo* fOCT mapping of the cilia location (shown in green) and confocal immunofluorescence imaging of the corresponding areas of the oviduct at the anterior ampulla close to osteum (**b**), the posterior ampulla close to isthmus (**c**) and the isthmus (**d**). Immunofluorescence staining shows beta tubulin (green) labeling the microtubules of the cilia, phalloidin (red) labeling the oviduct wall, and DAPI (blue) labeling the nuclei. Scale bars correspond to 100 μm. The schematic in (**a**) was created by the authors using Adobe Illustrator software.

To validate our fOCT findings, we compared the mapping of cilia locations using fOCT with confocal fluorescence microscopy of immunostained oviduct sections. The oviduct of a mouse at 0.5 days post-conception (dpc) was imaged *in vivo* with fOCT. Three locations were selected along the oviduct for the analysis: the anterior ampulla close to the osteum, the posterior ampulla close to isthmus, and the isthmus (Fig. 3a). Right after the fOCT imaging, the oviduct tissue was carefully dissected out and prepared for immunostaining. The mapping results from both fOCT and immunostaining clearly showed the decrease of the cilia coverage in the ampulla from the osteum side to the isthmus side (Fig. 3b,c) with isthmus having much fewer cilia than the ampulla (Fig. 3d). These findings correlate with previous *in vitro* observations in the human^46^ and mouse oviduct^47^. The distribution of the cilia in the lumen of the oviduct obtained by fOCT is in good agreement with the corresponding histological immunostaining (Fig. 3b,c), which is a widely-used standard cilia analysis method^17^,^18^,^19^. Quantitative analysis shows similar percentage of the cilia coverage of oviduct lumen obtained from fOCT and immunofluorescence histological analysis (see Supplementary Figure S1). These results confirm that the described fOCT approach allows for efficient mapping of the cilia location in the mouse oviduct *in vivo*.

### Validation of fOCT mapping of CBF

**Figure 4:**
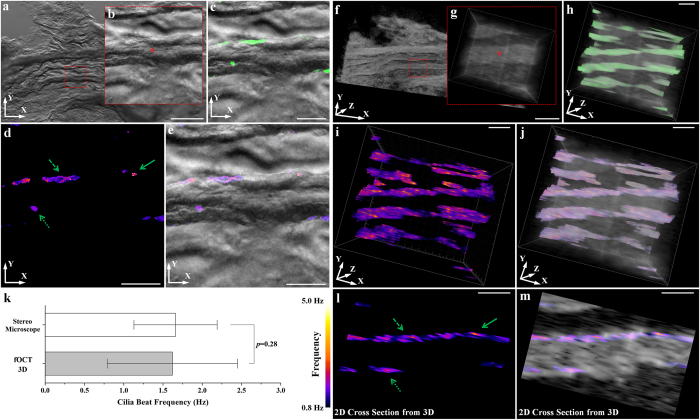
Validation of fOCT mapping of CBF. Images (**a**–**e**) correspond to bright-field microscopic analysis of CBF; Images (**f**–**m**) correspond to the fOCT analysis of the same area. (**a**) Bright-field microscopic image (low magnification) of the inner lumen of the ampulla from extracted mouse oviduct. The red dashed square indicates the analyzed region shown in (**b**). The star in (**b**) labels the major groove used in the analysis. (**c**) Mapping of the cilia location obtained from the time series of the microscopic images. (**d**) Corresponding mapping of CBF obtained from the microscopic images. (**e**) CBF mapping overlapped with the bright-field microscopic image. (**f**) 3D OCT structural image of the same area. The red dashed square indicates the same region of interest shown in (**g**). The star labels the same position of the groove. (**h**) fOCT mapping of the cilia location in 3D. (**i**) Corresponding fOCT mapping of the CBF in 3D. (**j**) 3D CBF mapping overlapped with the OCT structural image. (**k**) Statistics of the measured CBF from the bright-field microscopy and the 3D fOCT indicating the statistically insignificant difference from a two-sample two-tailed student’s *t* test with the alpha value of 0.05. Data are presented as mean ± standard deviation. (**l**,**m**) 2D cross-section images obtained from (**i**) and **(j**), respectively. Three different types of arrows are used to indicate the correspondence of the CBF mapping between fOCT and the bright-field microscopy in (**d**) and **(i**). Scale bars in (**a**) and (**f**) correspond to 300 μm. Scale bars in all other panels correspond to 50 μm.

To validate the acquired CBF measurements, we performed fOCT analysis as well as bright-field microscopy on an *in vitro* explanted oviduct tissue that was cut and opened up to expose the luminal surface in 1x phosphate buffered saline (PBS) at room temperature. The oviduct was first imaged using bright-field microscopy with a digital camera, a traditional imaging method that has been extensively utilized in the studies of CBF in the mammalian oviduct^11^,^12^,^13^,^14^,^15^,^41^,^42^. From the wide-field microscopic image (Fig. 4a), we focused on a typical region of a mucosa fold (Fig. 4b) and performed continuous 2D image recording with a frame rate of 51 Hz, which enables direct visualization of the cilia beat along the fold. Similar to standard CBF analyzing methods based on the intensity of the microscopic image^13^,^14^,^15^,^16^,^17^,^25^,^26^, fast Fourier transform with the spectral resolution of ~0.05 Hz was performed on each pixel of the image followed by mapping of the peak amplitude from the frequency spectrum, generating an image of the cilia locations (Fig. 4c). The frequency positions of the peaks in the spectrum were then mapped to the locations of cilia, providing the map of CBF (Fig. 4d,e). Right after imaging with the bright-field microscope, the same oviduct tissue was immediately transferred to the OCT imaging stage. A 3D OCT structural image (Fig. 4f) was first taken to identify the region of interest (Fig. 4g) matching the one from the optical microscope. Then fOCT imaging and analysis was performed in 3D that covers the target region, showing the cilia locations and the CBF mapped to the 3D oviduct structural image (Fig. 4h,i,j). A comparison of the 3D fOCT results with the CBF mapping from the bright-field microscope demonstrated that the frequency values obtained from these two techniques were similar. Images acquired with fOCT in Fig. 4h–j belong to the *en face* view of the 3D visualization over the whole imaging depth and, therefore, reveal larger ciliated areas, in contrast to the bright-field images in Fig. 4c–e, which show visualization of cilia only in a single focal plane of ~9 μm in thickness. The statistics of the CBF (Fig. 4k) measured with these two imaging techniques from the fields of view shown in Fig. 4d,i indicated no significant difference with a *p* value of 0.28 from a two-sample two-tailed student’s *t* test. This result further demonstrated that the fOCT technique is able to measure the CBF with the accuracy comparable to the widely-used *in vitro* approach. The tomographic cross-sectional image (Fig. 4l,m) selected from the fOCT 3D volume showed good agreement with the results from the bright-field microscopy, demonstrating the feasibility of mapping CBF using fOCT. It is important to note that the overall CBF measured *in vitro* with the oviduct dissected out and the ciliated surface exposed for imaging appears to be lower compared with the *in vivo* experiments (described below). Such difference can be explained by the effects of lower temperature, difference in local microenvironment, and interruption in the nutrient delivery, all of which could affect cilia dynamics^21^,^48^,^49^.

### *In vivo* fOCT imaging and studies of ciliary activity

**Figure 5:**
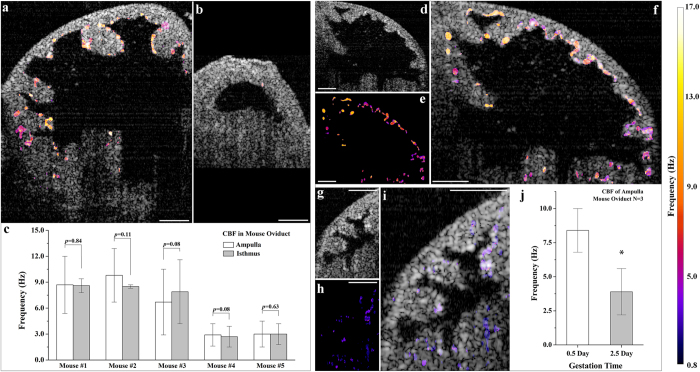
*In vivo* fOCT mapping of CBF in the mouse oviduct. (**a**,**b**) Typical *in vivo* fOCT mapping of CBF from the ampulla and the isthmus, respectively, in the same animal. (**c**) *In vivo* CBF measurements from mouse oviducts (number of mice n = 5) indicating the insignificance of the difference between the frequency of cilia beat from the ampulla and the isthmus (two-sided Wilcoxon rank-sum tests with the alpha value of 0.05). Data are presented with mean ± standard deviation. (**d**–**f**) Typical *in vivo* OCT structural image (**d**), fOCT mapping of the CBF (**e**), and overlapped image (**f**) from the mouse ampulla at 0.5 dpc. (**g**–**i**) Typical *in vivo* OCT structural image (**g**), corresponding fOCT mapping of the CBF (**h**), and the overlapped image (**i**) from the mouse ampulla at 2.5 dpc. (**j**) Statistics of *in vivo* CBF measurements showing higher frequency of the cilia beat from the mouse ampulla at 0.5 dpc in comparison with 2.5 dpc (*p *= 0.027 from a two-sample two-tailed student’s *t* test with the alpha value of 0.05). Data are presented with mean ± standard deviation. Scale bars correspond to 100 μm.

We next tested the feasibility of the described approach for *in vivo* investigations of the cilia activities in the mouse oviduct at different locations of the oviduct through different post-conception stages. Comparative fOCT analysis in the ampulla (Fig. 5a) and the isthmus (Fig. 5b) revealed similar CBF ranges. It is interesting to note that cilia are organized in patches of different sizes and the cilia from neighboring areas beat at distinct frequencies. A similar phenomenon has previously been observed *in vitro*^13^. Based on five mice used in this study, no significant difference has been detected (*p* values larger than 0.05) between the CBF from the ampulla and the isthmus of the same oviduct (Fig. 5c), suggesting that the cilia beats from these two different anatomical locations are likely to have the same CBF range. Such findings correlate with the *in vitro* experimental results of the CBF in the human fallopian tube^41^. We also performed comparative fOCT analysis on the posterior ampulla of the oviduct (close to the isthmus) at the days of 0.5 and 2.5 post-conception. Our measurements revealed that the frequency range of the cilia beat in the ampulla at 0.5 dpc (Fig. 5d,e,f) is higher than that at 2.5 dpc (Fig. 5g,h,i) with *p *= 0.027, less than 0.05 (alpha value) from a two-sample two-tailed student’s *t* test. The average CBF value over the imaging field of view from individual mouse was averaged (Fig. 5j) and corresponds to 8.4 ± 1.6 Hz at 0.5 dpc and 3.9 ± 1.7 Hz at 2.5 dpc (mean ± standard deviation). These results indicate that the described fOCT approach allows measuring a range of physiological CBFs and differentiating the frequencies at different post-conception stages in the mouse oviduct *in vivo*.

## Discussion

We present and validate an approach for live mapping of cilia and their dynamics in the intact mouse oviduct. *In vivo* capability and the large depth of view (covering the whole cross-section of the mouse oviduct tube) are two major advantages of the described approach over the existing widely-used microscopic methods that require opening up of the dissected oviduct and exposing the ciliated surface for imaging. In addition, unlike fluorescence imaging methods, fOCT relies on the natural optical contrast of tissues and does not require any exogenous contrast agents or vital reporters, which could possibly affect ciliary function and motility^50^. These important features of fOCT make it a unique research tool that opens the door for a number of *in vivo* studies in reproductive medicine and cilia biology in general.

The measurable frequency range of fOCT is determined by the sampling frequency of the OCT system. In the presented study, we applied a 100 Hz frame rate, resulting in 50 Hz of the highest detectable frequency, sufficient for the measurement of CBF in the mammalian oviduct^12^,^13^,^14^,^15^,^41^,^42^. Wider dynamic range is possible with faster acquisition of the B-scan OCT images. However, this generates larger data sets and complicates data storage and analysis. Based on a fixed frame rate, the pixel resolution in the frequency domain is dependent on the time duration of recording of the dynamic activity. With 10.24 seconds, we have obtained spectral resolution close to 0.1 Hz in our CBF measurement. Longer data acquisition for each frame location will result in smaller pixel size in the frequency domain. These settings could be optimized for different purposes during the fOCT imaging, such as minimizing the imaging time or improving the frequency resolvability. In terms of the spatial scale for the CBF mapping, we obtained ~2 μm in both the axial and the transverse directions with a depth of view able to cover the ampulla of the mouse oviduct. Employing an ultrahigh-resolution OCT system and increasing scanning positions during the fOCT imaging can further improve the spatial scale.

Our studies revealed lower CBF in the oviduct at 2.5 dpc in comparison to 0.5 dpc (Fig. 5j). While ciliary dynamics in the oviduct through preimplantation stages are not well characterized, the difference in CBF at different stages is not unexpected. It takes about four days for the oocyte to travel through the length of the oviduct, as it undergoes fertilization, transitions to zygote and undertakes first divisions. These dramatic transitions during the transport of the embryo through different compartments of the oviduct are associated with changes in hormonal levels, some of which can have an effect on cilia dynamics^12^,^42^. Sperm also have an effect on cilia dynamics^14^. At about 0.5 dpc, the motile sperm are present in the ampulla^51^, while at 2.5 dpc the sperm lose their motility^52^. Previous *in vitro* studies show that the presence of motile sperm in rat oviduct increases the CBF through adrenomedullin (ADM) hormone, which causes CBF increase^14^. Thus, the detected higher CBF of the ampulla at 0.5 dpc might be triggered by the presence of the motile sperm, which are losing their motility and getting cleared at 2.5 dpc. This question can be further investigated using the described *in vivo* fOCT approach in correlation with the analysis of hormonal levels. Comparative analysis of CBF in plugged and unplugged animals might provide an insight into *in vivo* regulation of the cilia dynamics by sperm.

Previous work has demonstrated that structural OCT imaging could be used to characterize microfluidic-scale flow generated by the motile cilia from the epithelium of *Xenopus tropicalis* embryos through tracking of introduced polystyrene microspheres^34^,^35^, which reveals the function of the cilia in an indirect way. This particle tracking velocimetry approach can potentially be combined with our fOCT technique in the mouse oviduct to study the transport of the oocytes and the cumulus cells through the oviduct in correlation with cilia function *in vivo*, investigate the fluid dynamics, and further understand the interplay between cilia function and biomechanical environment.

Potentially this imaging technique could be utilized in longitudinal studies of the development of mouse oviduct and ciliary functions. In the production of transgenic mice, mouse reproductive organs are routinely exposed by surgical manipulations for transferring zygotes^43^, which is not associated with any reproductive consequences. As our *in vivo* fOCT approach follows this procedure, we do not expect any detrimental effect from the imaging process. However, for prolonged imaging sessions, measures should be taken to prevent tissue dehydration and control the environmental conditions. Additional optimization procedures might be necessary to improve the consistency of the measurements and the tissue preservation depending on the purpose of the experiments.

Currently the role of cilia in fertility has not been well characterized, and the cilia dynamics in relation to fertilization and oocytes transport remain unclear^53^, largely due to the lack of powerful *in vivo* imaging tools. The described fOCT approach for *in vivo* CBF mapping might provide spatially-resolved information of cilia dynamics through estrous cycle and gestational stages. In correlation with structural analysis, oocyte tracking and molecular genetic analysis of pathways implemented in fertility, this approach can contribute to screening of pharmacological agents affecting motility and understanding of a variety of questions in reproductive biology.

Because the motile characteristic of the cilia is essential for various biological processes in the mammalian body^6^, applications of the reported fOCT are not limited to the assessment of oviduct ciliary function. For example, the motility of cilia at the ventral surface of the embryonal node in mammals generates flow that is necessary for initiating the signaling cascade for left-right patterning during development^54^. Since OCT has been successfully applied for live imaging of post-implantation mouse embryos^55^, the fOCT method could be directly implemented to provide advanced phenotyping for related research in developmental biology. In addition, the integration of this fOCT CBF mapping approach with the endoscopic probe^56^ could lead to *in vivo* simultaneous depth-resolved functional and structural assessment of ciliated epithelial surfaces in the body, such as the human fallopian tube and respiratory tract. Therefore, the fOCT technique introduced in this paper has potentially broad applications in cilia-related biological studies and clinical diagnosis.

## Methods

### Optical coherence tomography system

We utilized a home-built spectral domain optical coherence tomography (OCT) system with a Titanium:Sapphire laser (Micra-5, Coherent, Inc) providing low-coherence light with the bandwidth of ~110 nm centered at ~808 nm. The collimated laser beam is coupled into a fiber-based interferometer with a neutral density filter to control the light intensity. The interference of the light from the reference and the sample arms is spatially separated in terms of the wavelength and focused onto a high-speed line scan CMOS camera (spL4096-140 km, Basler, Inc) with the spectral resolution of ~0.029 nm. With fast-Fourier transform of the equal *k*-space fringes, the 4096 pixels of the camera enables 2048 spatial resolving points of A-scan for the imaging depth of ~5.6 mm available in air. Two galvanometer-mirrors were used to obtain the transverse scanning of the laser beam across the sample. The control and synchronization of the mirror movement is realized with signals generated from the computer. The OCT system provides an axial resolution of ~5 μm in tissue with the imaging beam diameter of ~4 μm at the focal plane.

### Immunohistochemistry and fluorescence imaging

After dissection, the oviduct tissue was embedded in optimal cutting temperature compound and immediately transferred to −30 °C storage. The frozen volume was sectioned at ~10 μm and fixed in 4% methanol-free paraformaldehyde in phosphate-buffered saline (PBS). The sections were first washed with PBS (5 minutes) three times, and then permeabilized with PBS containing 0.1% Triton X-100 (10 minutes) repeated three times at room temperature (RT). After this, PBS was used again to wash the sections (5 minutes) three times. The sections were then incubated at RT with 5% bovine serum albumin (BSA) in PBS for at least one hour. After this, tissue sections were incubated with the primary antibody anti-beta tubulin (1:400 dilution) in PBS containing 5% BSA overnight at 4 °C. Sections were then washed with PBS (5 minutes) three times. After this, sections were incubated with the secondary antibody DyLight 488 conjugated rabbit IgG (H&L) (1:400 dilution), CF594 conjugated phalloidin (1:80 dilution), and diamidino-2-phenylindole (DAPI; 1:100 dilution) in PBS containing 5% BSA for at least one hour at RT. Sections were then washed (5 minutes) three times, mounted, and covered with micro cover glasses. The processed specimens were kept at 4 °C. Here, the rabbit IgG (H&L) antibody is used to label the anti-beta tubulin antibody that targets the microtubules of the cilia, the phalloidin reacts with F-actin and labels the cytoskeleton of the cells, and the DAPI stains the nucleus of the cells. The anti-beta tubulin antibody is purchased from Abcam plc. (ab6046). The rabbit IgG (H&L) secondary antibody with DyLight 488 conjugated is purchased from Rockland Immunochemicals Inc. (611-741-127). The CF594 conjugated phalloidin is purchased from Biotium Inc. (00045-300U).

Imaging of the stained oviduct sections was conducted using a confocal laser scanning microscope (Zeiss LSM 780) with a 20x plan-apochromatic dry objective (0.8 NA). Both solid-state and Argon lasers were utilized for the fluorescence imaging, which provide 561 nm light for the excitation of CF594, 488 nm for the excitation of DyLight 488, and 405 nm for the excitation of DAPI. The corresponding filters of these three channels select the wavelength ranges of 579–685 nm (CF594), 499–570 nm (DyLigh 488) and 410–495 nm (DAPI) for visualization. The diameter of the pinhole was set to be 27 μm for each channel.

### Bright-field microscopic imaging

Bright-field microscopic imaging of the *in vitro* oviduct was performed using a Zeiss Axio Zoom. V16 microscope equipped with a digital camera (AxioCam HRm). The time series of the bright-field microscopic images from the oviduct were taken with 180x magnification at a frame rate of 51 Hz. The acquired 2D images have the pixel scale of 1.15 μm for both transverse directions. The depth of field provided by the microscope is ~9 μm.

### Mouse manipulation

The wild type CD-1 male and female mice were paired to establish timed mattings overnight as determined by the presence of a vaginal plug. The presence of the plug in the day is designated as 0.5 days post-conception (dpc). At 0.5 dpc or 2.5 dpc, mice were anesthetized by intraperitoneal injection with a 1.25% Avertin solution and placed on a heating pad at 37 °C to maintain body temperature. Hair removal cream was used to expose the dorsal lateral skin. The depilated skin was treated with 70% ethanol and a small incision (~1 cm) was made parallel to the dorsal midline. A blunt forceps was used on the ovarian fat pad to gently pull the ovary/oviduct/uterus out of the body. A vessel clamp was utilized to hold the fat pad to keep the exposed tissues stable for imaging. The vessel clamp was fixed onto a stabilized optical breadboard, which helped to avoid motion artifacts due to breathing and heartbeat. Adjustments of the whole tissue structure with forceps were performed under dissection microscope to orient the oviduct facing up for imaging with the OCT system. The whole process is conducted at room temperature. The animals were sacrificed after the imaging and the oviducts were collected for subsequent histological analysis. All animal manipulation procedures described here were approved by the Animal Care and Use Committee of the Baylor College of Medicine. All experiments were carried out in accordance with the approved guidelines and regulations.

### Software

For fOCT imaging, the OCT system control and data acquisition were through LabVIEW (National Instruments Corporation). For bright-field imaging and confocal imaging, ZEN software provided by Zeiss was used to capture the images. The data processing described in the paper was performed using Matlab (MathWorks, Inc.). Imaris software (Bitplane) was used for image registration and 3D rendering.

## Additional Information

**How to cite this article**: Wang, S. *et al*. *In vivo* micro-scale tomography of ciliary behavior in the mammalian oviduct. *Sci. Rep*. **5**, 13216; doi: 10.1038/srep13216 (2015).

## Supplementary Material

Supplementary Information
