# Supplementary material for: In vivo micro-scale tomography of ciliary behavior in the mammalian oviduct
Source: Sci Rep. 2015 Aug 17;5:13216. doi: 10.1038/srep13216 (PMC4538602; doi:10.1038/srep13216)
Supplement: Supplementary Information [file srep13216-s1.pdf]

Supplementary Information

In vivo micro-scale tomography of ciliary behavior in the mammalian oviduct

Shang Wang[1,3], Jason C. Burton[1,3], Richard R. Behringer[2] & Irina V. Larina[1,*]

[1]Department of Molecular Physiology and Biophysics, Baylor College of Medicine, Houston, TX 77030, USA

[2]Department of Genetics, University of Texas M.D. Anderson Cancer Center, Houston, TX 77030, USA

[3]These authors contributed equally to this work

*Correspondence and requests for materials should be addressed to I.V.L (larina@bcm.edu)

Contents:

Supplementary Figure S1: Page 2

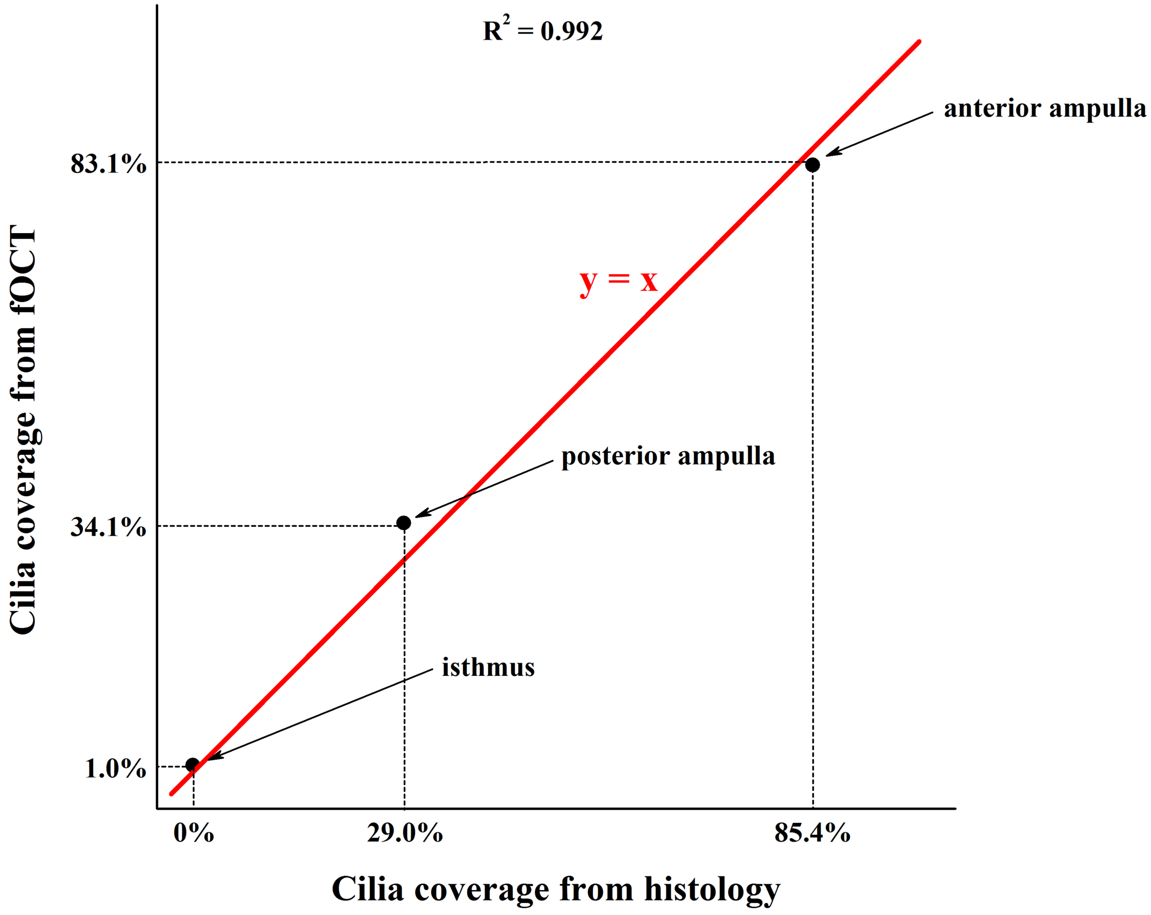

Figure S1: Estimations of the cilia coverage of the oviduct inner lumen show similar cilia mapping results between fOCT and corresponding immunohistochemical staining. The graph represents the quantitative comparison of the luminal cilia coverage revealed by fOCT and corresponding immunohistochemistry based on the images presented in Figure 3. The lumen of the oviduct was manually traced, and the percentage of the pixels that are shown as ciliated was used as the measure of cilia coverage. The data points for three distinct locations along the oviduct are plotted with the ideal y=x line. The R-square value of 0.992 indicates that the percentages of the luminal cilia coverage revealed by fOCT are similar to the ones from histological immunostaining analysis of corresponding areas.
